# Accuracy of Glucose Meter among Adults in a Semi-urban Area in Kathmandu, Nepal

**DOI:** 10.31729/jnma.4247

**Published:** 2019-04-30

**Authors:** Asmita Pokhrel, Vinutha Silvanus, Buddhi Raj Pokhrel, Binaya Baral, Madhav Khanal, Prajwal Gyawali, Laxman Pokhrel, Deepak Regmi

**Affiliations:** 1 Department of Biochemistry, Nepal Medical College and Teaching Hospital, Kathmandu, Nepal; 2 Department of Community Medicine, Nepal Medical College and Teaching Hospital, Kathmandu, Nepal; 3 Department of Biochemistry, Universal College of Medical Sciences, Bhairahawa, Nepal; 4 School of Biomedical Sciences and Pharmacy, University of New Castle, NSW, Australia; 5 Nepal Medical College and Teaching Hospital, Kathmandu, Nepal

**Keywords:** *accuracy*, *analytical*, *Bland-Altman plots*, *clinical*, *glucose meter*, *Park error grid*

## Abstract

**Introduction:**

Glucose meters are gaining popularity in monitoring of blood glucose at household levels and in health care set-ups due to their portability, affordability and convenience of use over the laboratory based reference methods. Still they are not free of limitations. Operator's technique, extreme temperatures, humidity, patients' medication, hematocrit values can affect the reliability of glucose meter results. Hence, the accuracy of glucose meter has been the topic of concern since years. Therefore, present study aims to evaluate the analytical and clinical accuracy of glucose meter using International Organization for Standardization 15197 guideline.

**Methods:**

A community based descriptive cross-sectional study was conducted in Kapan, Kathmandu, Nepal in April 2018. Glucose levels were measured using glucose meter and reference laboratory method simultaneously among 203 adults >20 years, after an overnight fasting and two hours of ingestion of 75 grams glucose. Modified Bland-Altman plots were created by incorporating ISO 15197 guidelines to check the analytical accuracy and Park error grid was used to evaluate the clinical accuracy of the device.

**Results:**

Modified Bland-Altman plots showed>95% of the test results were beyond the acceptable analytical criteria of ISO 15197:2003 and 2013. Park Error Grid-Analysis showed 99% of the data within zones A and B of the consensus error grid.

**Conclusions:**

Glucose meter readings were within clinically acceptable parameters despite discrepancies on analytical merit. Possible sources of interferences must be avoided during the measurement to minimize the disparities and the values should be interpreted with caution.

## INTRODUCTION

Glucose meters are gaining popularity in monitoring of blood glucose at household levels, in health care set-ups due to their portability, affordability and convenience of use over the laboratory based reference methods.^1,2^ Still they are not free of limitations. Operator's technique, extreme temperatures, humidity, patients' medication, hematocrit values can affect the reliability of glucose meter results.^3,4^

Ever since the introduction of glucose meters in market for home use during 1980s, accuracy always has been the topic of clinical concern everywhere.^5^ Numerous international studies performed on the accuracy of glucose meters have shown conflicting results,^6–8^ and even many glucose meter systems cleared by Food and Drug Administration did not satisfy the minimal accuracy criteria.^6^ Such information could be helpful to the patients and health care professional while working with the glucose meters.

Our study attempts to evaluate the analytical and clinical accuracy of glucose meter in Nepalese community setting using International Organization for Standardization (ISO) 15197 guideline.

## METHODS

A community based descriptive cross-sectional study was conducted on seven different days in a month time in Kapan, a semi-urban area of Kathmandu in April 2018 among adults 20 years. Ethical approval was obtained from Nepal Medical College Institutional Review Committee. An informed verbal consent was taken from the participants and participation was voluntary. Acutely ill participants, pregnant women and those below 20 years were excluded from the study. Demographic and medical details were noted. A minimum sample size of 143 was calculated using formula:


$$\text{Sample size (n)}={\text{Z}}^{2}{\text{pq/d}}^{2}$$

Where,

- Z = 1.96 for 95% confidence interval,
- p = prevalence of diabetes mellitus 8.4%,^9^
- d = margin of error 5%,
- 20% non-response rate was assumed.

Capillary (finger-prick) and venous blood samples for the glucose estimation by glucose meter and reference laboratory method was carried out for fasting and two hour Oral Glucose Tolerance Test (OGTT). Participants were given to drink 75 grams of glucose dissolved in 250–300 ml of water over a period of five minutes. Two hour postprandial tests was offered to the 41 participants with known history of diabetes. Out of 203 participants participated, only 107 agreed for second capillary pricks. Three ml of venous blood was collected from ante-cubital vein under aseptic condition in fluoride containing tubes for plasma glucose estimation. Collected venous blood samples were stored in a lab specimen transport bag till processed further. The venous samples were transported to laboratory, separated to plasma and glucose was estimated using fully automated analyzer Johnson & Johnson Vitros 250, USA, in Nepal Medical College and Teaching Hospital (NMCTH), Jorpati, Kathmandu. Two trained laboratory staffs were recruited for venipuncture and two glucose meters of same brand were used for the capillary glucose testing throughout the research program.

Glucose meters were cleaned and disinfected with 70% isopropyl alcohol for 5 minutes after each use to assure the safety of the participants. The used sharps were collected in the sharp container and were disposed as per the waste disposal protocol of the NMCTH.

The glucose meter being used is based on glucose oxidase system and is calibrated to display plasma-like concentration results. The range of measurement is 0.633.3 mmol/L and it measures glucose concentration in a 0.5 /vl sample of whole blood.

Data was analysed using Stata15IC licensed software. Modified Bland- Altman (BA) plots were created by incorporating ISO 15197:2003 and 2013 accuracy guidelines to evaluate the analytical accuracy of glucose meter.^10,11^ The difference between reference method results and glucose meter system was plotted on the y-axis, with reference results plotted on the x-axis.

Regarding minimum accuracy criteria, ISO 15197:2013 stipulates that at least 95% of measurement results shall fall within±15 mg/dl of the reference value at blood glucose (BG) concentrations<100 mg/dl and within±15% at BG concentrations>100 mg/dl and at least 99% of measurement results shall fall within the Consensus Error Grid zones A and B. Thus, accuracy criteria are more stringent than in ISO 15197:2003 which stipulated 95% of glucose meter readings should be within±15 mg/dl at BG concentrations<75 mg/dl and ±20% at BG concentrations>75 mg/dl^12^ the ISO (International Organization for Standardization

Type 2 diabetes version of Park Error Grid analysis (PEG-A) was used for the assessment of clinical accuracy of the test results. The PEG specifies five risk levels and is divided into 5 risk zones as A, B, C, D and E. Definition of different risk zones are: A-clinically accurate measurements, no effect on clinical action, B-altered clinical action, little or no effect on clinical outcome, C-altered clinical action, likely to affect clinical outcome, D-altered clinical action, could have significant clinical risk, E-altered clinical action, could have dangerous consequences.^13^

## RESULTS

A total of 203 participants (79 male, 124 female) from the community participated in the study. There were 41 participants with known history of diabetes and were under medication. Mean age of the study participant was 50.97±15.12 years, ranging from 21 to 87 years.

**Figure 1.**
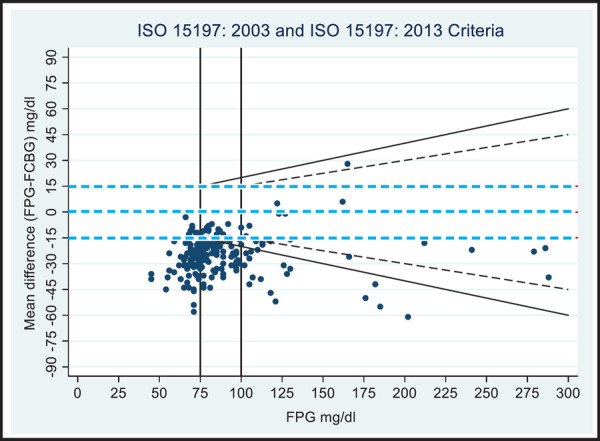
Modified Bland Altman plot of fasting glucose values.

**Figure 2.**
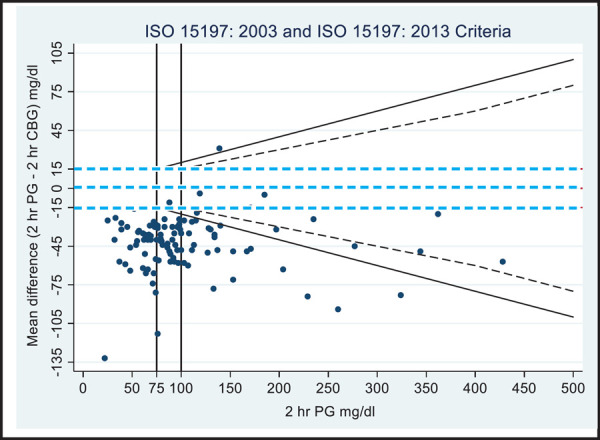
Modified Bland Altman plot of 2 hour glucose values.

Figure 1 and 2 show modified BA plots with incorporated ISO 15197:2000 and 2013 accuracy guidelines. When the glucose meter readings of fasting were evaluated according to ISO 15197:2013 criteria, 22 of 165 (13.3%) results were found to be within15 mg/dl for fasting plasma glucose (FPG)< 100 mg/dl and 11 of 38 (28.9%) results were within15% for fasting FPG>100 mg/dl. Whereas, according to ISO 15197:2003 criteria, 8 out of 70 (11.4%) results were found to be within 15 mg/dl for FPG values<75 mg/dl and 16 of 133 (12%) results were within20% for FPG>75 mg/dl (Figure 1). Similarly, more than 95% of the 2 hour glucose results too were beyond the expected range (Figure 2).

**Figure 3.**
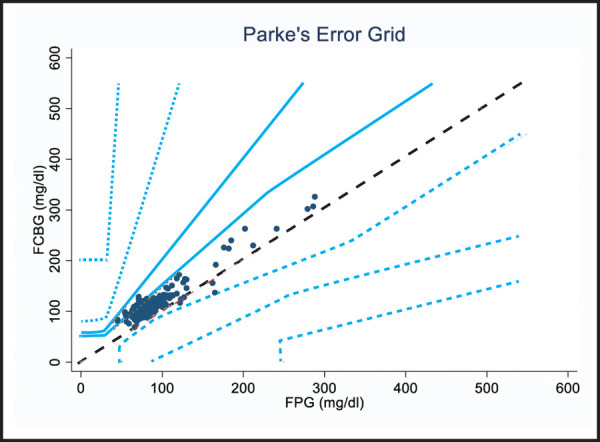
PEG-A of type 2 diabetes version.

PEG-A analysis represents the distribution of glucose results estimated by glucose meter versus that by reference method. The dotted line shows exact agreement between the two i.e. glucose meter and reference method. More than 99% of the data were within zones A and B of consensus error grid (Figure 3).

## DISCUSSION

According to PEG-A, 99% of our results are within zones A and B which reflects that our glucose meter results are clinically accurate with little or no effect on clinical outcome (Figure 3). This finding is consistent with the findings of another recent study done among pediatric patients by Rojekar MV et al.^4^

Despite the test results being clinically accurate according to the ISO 15197:2013 guideline as demonstrated by PEG-A, analytically, the data points do not meet the criteria specified by both 2003 and 2013 guidelines. More than 5% of data are outside the cut-off points (Figures 1 and 2). The ISO-criteria is more about analytical accuracy rather than clinical accuracy. Some of the analytically inaccurate data points might still be clinically acceptable. This might be the reason for introducing PEG as an accepted evaluation tool in new draft of ISO 15197:2013 guideline.

Likewise, in the present study, glucose meter readings are beyond the acceptable criteria analytically when compared with reference method readings. A number of physiological and technical factors may account for this discrepancy seen. Apart from the technical specification of the instrument, approximately 91–97% of overall inaccuracies are operator dependent^14^ though the glucose meter device is considered to be less technique sensitive. Nevertheless, it should be noted that in the present study, pre-analytical errors related to sample like presence of bubbles or clots and inadequate sample were tried to be minimized. Trained phlebotomist and glucose meter operators were recruited for performing the procedure. However, in real practice, in resource strained country like Nepal, there is no such practice of training on glucose meter operations.

Improper storage and prolonged exposure of strips to extreme temperature, humidity and moisture also account for the discrepancies.^15^ Possible explanation for such a discrepancy in the test results could be due to the delay in the sample transportation from the community to the laboratory, hence giving rise to pre-analytical error. The venous samples though were collected in tubes containing sodium fluoride, centrifugation and plasma separation was delayed. American Diabetes Association has recommended immediate separation of sample to plasma or the sample should be collected into a container with glycolytic inhibitors and placed in ice-water until separated prior to analysis.^16^ However, it takes around 1–2 hours for the fluoride to get across into the red blood cell^17^ and the glucose levels reduces by 5–7% every hour due to consumption by glycolysis. ^18^

Use of glucose meters are not only limited to diagnosed diabetes patients, they are widely and regularly used in many other areas of health care, such as in hospital's emergency, intensive care units, wards, physician's offices, in emergency response units, during dialysis, in aged care facilities, and by rescue services.^19^ Though, in the present study conducted among community participants, the discrepancies in the results among two methods did not affect clinical decision making, this might not be the case in the hospital settings. There is controversy regarding the performance of glucose meter in the care of critically ill patients.^20–23^ Disagreement among different measures of glucose was reported in the critical care set-up, causing trouble in clinical decision making regarding insulin infusion protocol for aggressive glucose control.^24^ A wide source of interferences needs to be considered while interpreting glucose meter results.^15^ An extreme physiological status like stress, oxygenation, perfusion, blood pH, medications, serum triglycerides, uric acid, and para protein levels might play a significant source of bias in hospital set-up.^21,22,24^ Limitations of our study were that the blood samples could not be separated and analysed immediately and the hematocrit values of the participants was not considered.

## CONCLUSIONS

The present study concluded that although the glucose meter readings were found to have no effect on the clinical decision making and outcome, there seems to be a significant gap in the analytical performance of glucose meters compared to the standard laboratory based analyser. Potential sources of interferences must be minimized while using the glucose meters and the values obtained should be interpreted with caution. Further studies are highly recommended and the development of glucose meters that along with the clinical accuracy also satisfy the ISO 15197:2013 criteria of analytical accuracy is awaited.
